# Serum and salivary levels of lactate dehydrogenase in oral squamous cell carcinoma, oral lichen planus and oral lichenoid reaction

**DOI:** 10.1186/s12903-020-01306-0

**Published:** 2020-11-10

**Authors:** Narges Gholizadeh, Maryam Alipanahi Ramandi, Maziar Motiee-Langroudi, Mehrdad Jafari, Hadi Sharouny, Nafiseh Sheykhbahaei

**Affiliations:** 1 Department of Oral and Maxillofacial Medicine, School of Dentistry, Tehran University of Medical Science, Tehran, Iran; 2 School of Dentistry, Tehran University of Medical Science, Tehran, Iran; 3 Otorhinolaryngology Research Center, Tehran University of Medical Sciences, Tehran, Iran

**Keywords:** l-lactate dehydrogenase, Lichen planus, Oral, Lichenoid eruptions, Carcinoma, Squamous cell, Saliva

## Abstract

**Background:**

Increased levels of lactate dehydrogenase (LDH) as a tumor marker have been reported in malignant and some premalignant oral lesions such as oral lichen planus (OLP) and oral lichenoid reactions (OLRs). This study aimed to assess the level of total LDH in the saliva and serum of patients with oral squamous cell carcinoma (OSCC), OLP and OLRs.

**Methods:**

In this case–control study, the participants were divided into four groups (n = 25) of healthy controls, OLP, OLRs, and OSCC. The serum and stimulated/unstimulated salivary levels of LDH were spectrophotometrically measured using standard LDH kits (Pars Azmoun). One-way ANOVA, Chi-square test, Pearson’s correlation test, and receiver operating characteristic (ROC) analysis were applied to analyze the data.

**Results:**

The serum and salivary levels of LDH in OSCC patients were significantly higher than that the corresponding values in other groups (*P* = 0.0001). The serum level of LDH in OLR group was significantly higher than that in the control and OLP groups (*P* = 0.0001), but the difference in salivary level of LDH was not significant. The ROC analysis showed that both the serum and salivary levels of LDH had significant diagnostic ability for detection of OSCC and OLRs. Significant associations were noted between the serum and salivary levels of LDH.

**Conclusions:**

Patients with OSCC and OLRs had higher serum levels of LDH than OLP and control groups. Further prospective longitudinal studies are required to assess the tissue level of LDH and monitor the transformation of OLRs because they have low rate of malignant transformation compared with other oral premalignant lesions.

## Introduction

Oral squamous cell carcinoma (OSCC) is the sixth most common cancer worldwide. OSCC is highly prevalent in developing countries due to certain risk profiles and difficult accessibility of health services. Males have higher incidence and mortality rates than females. Excessive tobacco use and alcohol consumption are the main risk factors for about 90% of oral cancers [1]. Delayed diagnosis is the main reason for high mortality rate of OSCC [2]. The process of malignant transformation may be stopped if potentially malignant oral lesions such as oral lichen planus (OLP) and oral lichenoid reactions (OLRs) are detected and treated early enough [3, 4]. The prevalence of OLP and OLRs in the general population is 1–2%, and 2.4%, respectively. Both OLP and OLRs are more frequent in women in their fifth decade of life. OLP is a common chronic immunological mucocutaneous disease with an unknown etiology [5, 6]. However, factors such as stress, genetics, and immunological factors can contribute to the development of OLP by induction of cell-mediated or auto-immune responses [7]. In contrast, OLRs, as a type IV hypersensitivity reaction, may occur as an uncommon cutaneous or mucosal adverse effect of dental materials, systemic drugs, or graft versus host disease [5]. OLP and OLRs are clinically and histologically similar to a great extent [8]. Typical clinical characteristics of the two conditions include the presence of white striae and small papules called the Wickham's striae, white plaque-like appearance, erythematous patches (atrophic/erosive), and/or ulceration of oral mucosa [9]. OLP is characterized by often symmetrical bilateral lesions that are typically widespread in the oral cavity. OLRs are often confined to the site in contact with the allergenic material. In drug-induced OLRs, there is a medical history of medication intake. The radiation form of striate with hyperpigmentation is more common in drug-induced OLRs but not for OLP. Involvement of uncommon sites such as the palate has been more commonly reported for OLRs and graft versus host disease [9, 10].

The main histopathological features of OLP include band-like infiltration of inflammatory cells in the superficial part of the connective tissue, hydropic degeneration of the basal layer keratinocytes, and loss of dysplasia [11]. Some discriminative microscopic features of OLRs from OLP include mixed inflammatory infiltration of plasma cells, neutrophils, and eosinophils, and a more diffuse and deeper pattern of infiltration in the connective tissue. Perivascular inflammation and higher number of Civatte bodies are in favor of OLRs [12].

The erosive, ulcerative [13–15], and plaque-type [16, 17] OLP have the highest risk of malignant transformation. Sufficient data regarding the risk of malignant transformation of different types of OLRs are not available, but it appears that different types of graft versus host disease and oral lichenoid contact lesions have higher risk of malignant transformation than drug-induced OLRs [18, 19]. The risk of cancer development at the site of chronic inflammation, and presence of inflammatory cells in the cancer tissue suggest the correlation of chronic inflammation and malignant transformation of lesions into oral cancer [20].

Despite easy access to the oral cavity for direct oral examination, oral malignancies are not often detected/diagnosed until late stages [21]. Thus, increasing attention has been paid to the role of tumor markers in management of the head and neck cancerous and potentially malignant oral lesions especially for diagnostic purposes [3].

Development of oral cancer has been linked to high glycolytic activity with a shift from aerobic glycolysis to anaerobic glycolysis. An increase in level of lactate dehydrogenase (LDH) may also occur along with increased glycolytic activity in neoplastic tissues of the thyroid, stomach and prostate [4, 22, 23]. LDH is a biomarker for cancer detection, which is found in almost all cell types [2]. Increased serum levels of LDH isoenzymes have been reported in different cancer types such as the lung, breast, cervical, nasopharyngeal, hematopoietic, and stomach cancers [3, 21, 24–30]. Moreover, increased level of this tumor marker has been reported in malignant and premalignant oral lesions such as leukoplakia, submucosal fibrosis, and OLP, compared with normal tissue [2, 21, 30, 31]. However, the level of LDH in OLRs has not been studied. Evidence shows that OLRs have higher rate of malignancy than OLP [20]. Many studies have measured the serum or tissue level of LDH isoenzymes in oral lesions [2, 3, 28, 29, 31]. The urinary LDH has also been used for detection of some disorders [32]. However, the salivary LDH alone or in combination with its serum level has been less commonly studied [2, 3, 25, 28–31]. Since saliva, as a non-invasive medium, is not yet routinely used for diagnostic purposes, further studies are required to determine the normal range and correlation of different biomarkers present in both serum and saliva such as LDH. Thus, this study aimed to assess the level of total LDH in the saliva and serum of patients with OSCC, OLP and OLRs as well as healthy controls to assess the efficacy of saliva sampling as a valuable tool and determine the correlation of serum and salivary LDH levels.

## Materials and methods

This case–control study evaluated 100 participants presenting to the Oral and Maxillofacial Medicine Department of Tehran University of Medical Sciences and patients hospitalized in the Cancer Institute and the ENT Department of Imam Khomeini Hospital. The participants were divided into four groups (n = 25) of healthy controls, OLP, OLRs, and OSCC. The participants were allocated to the study groups by consecutive sampling.

The minimum sample size was calculated to be 25 participants in each group according to a study by Patel et al. [21] and using the one-way ANOVA feature of PASS 11 software, assuming α = 0.05, β = 0.2, the mean standard deviation of 100.4, and effect size = 0.7.

The study protocol was approved by the Ethics Committee of TUMS (IR.TUMS.DENTISTRY.REC.1397.058). Written informed consent was obtained from the participants prior to their enrollment.

The inclusion criteria were confirmed cases of OSCC based on histopathological evidence, clinically and histopathologically confirmed cases of OLP according to the modified WHO criteria [11], clinically and histopathologically confirmed cases of OLRs, and healthy controls with no oral lesion. Patients with systemic conditions such as cardiac, hepatic, or renal disease, diabetes mellitus, other malignancies, substance abusers, pregnant women, those taking medications, patients with OLRs, OLP or OSCC under treatment, and patients with periodontitis or other mucosal lesions that could affect the LDH level were excluded.

Blood and saliva samples were obtained from the participants in all four groups. To prevent the effect of the circadian rhythm on the saliva flow, saliva samples were collected between 10 a.m. to 12 p.m. In order to collect unstimulated saliva samples, the participants were requested to refrain from eating and drinking for 60–90 min prior to sampling, and then they were asked to swallow their saliva in resting position, bend their head forward, and spit their saliva into graded sterile plastic vials. For collection of stimulated saliva, the participants were requested to chew equal pieces of mastic gum for 1 min and then spit it out, swallow their saliva, and spit into a Falcon tube. The saliva samples were then centrifuged, and pure saliva without sputum was transferred into microtubes. Following collection, saliva was immediately centrifuged at 2000 rpm for 10 min to remove squamous cells and cell debris. The supernatants were stored at − 20 °C until further analysis. Venous blood samples (5 cc) were drawn from the antecubital vein by a syringe and centrifuged at 2000 rpm for 10 min to separate the hematocrit from the plasma. Next, the samples were stored at − 20 °C in test tubes containing 3% citric acid as anticoagulant and sent to a laboratory within 2 h. The serum and salivary levels of LDH were spectrophotometrically measured within 24 h using standard LDH kits (Pars Azmoun, Tehran, Iran).

### Statistical analysis

Data were analyzed using SPSS version 24. One-way ANOVA was used to compare the raw data between the groups. The Chi-square test was applied to assess the correlation of parameters such as age, gender and tumor grade. The correlations between the groups were analyzed using the Pearson’s correlation test. Sensitivity of the assays was schemed against the false positivity using the receiver operating characteristic (ROC) curve. Comparison of area under the curve (AUC) was performed using a two-tailed *P* test. Accordingly, we measured the diagnostic power of the serum and salivary LDH levels for categorizing the disease status or patients (OSCC and OLRs vs. control).

## Results

This case–control study evaluated 100 participants including 34 males and 66 females. The four groups were matched in terms of gender with no significant difference. The mean age of participants was 42.73 ± 2.37 years in the control group, 49.73 ± 3.19 years in the OLP group, 52.73 ± 2.78 years in the OLR group and 61.00 ± 3.22 years in the OSCC group. The overall mean age of participants was 51.86 ± 1.60 years. The mean age of OSCC patients was higher than that of control and OLP groups; while, the mean age of OLR group was higher than that of control group (Table 1).

**Table 1:** Age and gender of participants in the four study groups

	Healthy individuals	OLP	OLRs	OSCC	*P* value
Age (years)	42.73 ± 2.378	49.73 ± 3.194	52.73 ± 2.784*	61.00 ± 3.225*^#^	0.000
Gender (M/F)	8/17	8/17	8/17	10/15	0.9

*^, #, $^A significant difference with the healthy control, OLP and OLR groups at *P* < 0.05, respectively

Table 2 shows the mean serum and salivary levels of LDH in the four groups. The highest mean level of LDH was noted in the OSCC group followed by the OLR, OLP, and control groups. The serum level of LDH was significantly higher than its salivary level in all groups.

**Table 2:** Serum and salivary levels of lactate dehydrogenase in the four study groups

Variable	Healthy individuals	OLP	OLRs	OSCC	*P* value
Serum LDH (u/L)	29.375 ± 6.4879	52.375 ± 14.7595	122.273 ± 16.6355*^#^	335.333 ± 41.1007 *^#$^	0.000
Unstimulated salivary LDH (U/L)	3.833 ± 1.1044	4.917 ± 1.3104	14.682 ± 3.0041	99.833 ± 49.3260*^#$^	0.021
stimulated salivary LDH (U/L)	3.500 ± 1.0751	3.638 ± 0.9776	20.909 ± 5.5424	112.208 ± 40.2209*^#$^	0.001

The data were expressed as mean ± standard error of the mean and analyzed by one-way ANOVA and Tukey’s post-hoc test, and *, # and $ indicate a significant difference with the healthy control, OLP and OLR groups at *P* < 0.05, respectively

The serum level of LDH in OSCC patients was significantly higher than that in the control, OLP, and OLR groups (*P* = 0.0001).

**Fig. 1:**
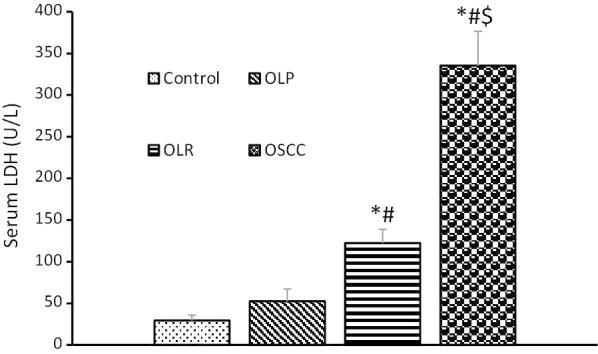
Serum levels of lactate dehydrogenase in the four study groups. *, # and $ indicate a significant difference with the healthy control, OLP and OLR groups at *P* < 0.05, respectively

The serum level of LDH in the OLR group was significantly higher than that in the control and OLP groups, and lower than that in the OSCC group (*P* = 0.0001). The serum level of LDH in OLP group was not significantly different from that in the control group (*P* > 0.05, Fig. 1).

**Fig. 2:**
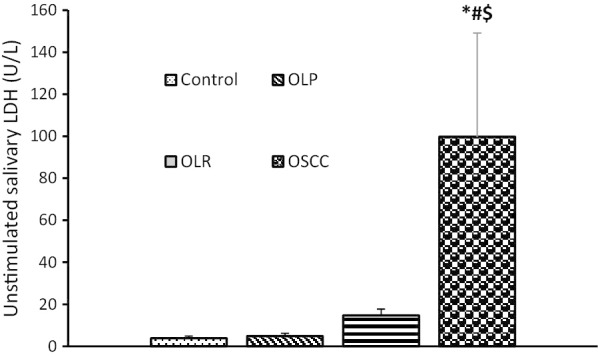
Unstimulated salivary levels of lactate dehydrogenase in the four study groups. *, # and $ indicate a significant difference with the healthy control, OLP and OLR groups at *P* < 0.05, respectively

**Fig. 3:**
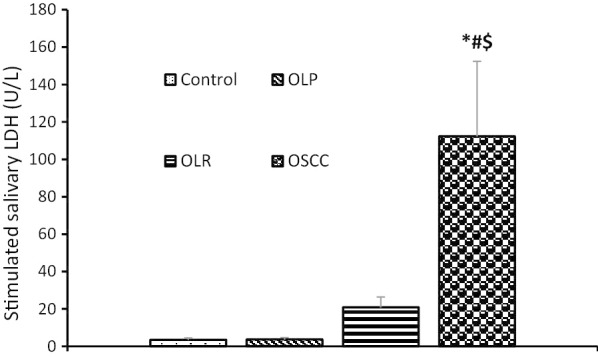
Stimulated salivary levels of lactate dehydrogenase in the four study groups. *, # and $ indicate a significant difference with the healthy control, OLP and OLR groups at *P* < 0.05, respectively

The salivary level of LDH (stimulated and unstimulated) in OSCC patients was significantly higher than that in the control, OLP and OLR groups. The salivary level of LDH (stimulated and unstimulated) in OLR group was higher than that in the control and OLP groups, but not significantly. The salivary level of LDH (stimulated and unstimulated) in the OLP group was almost similar to that in the control group (Table 2, Figs. 2, 3).

The serum level of LDH in grade 3 OSCC was significantly higher than that in grades 1 and 2 (*P* = 0.009). The serum level of LDH in certain types of OLR with a higher probability of malignancy (erosive and plaque types) was higher than that in the control and OLP groups but not significantly (*P* = 0.567).

The Pearson’s correlation test revealed a significant association between the serum level of LDH and its concentration in stimulated and unstimulated saliva samples, which indicated that by an increase in the serum level of LDH, its concentration in stimulated and unstimulated saliva also increased (Table 3).

**Table 3:** Correlation between the serum and salivary levels of lactate dehydrogenase

Variable	Unstimulated salivary LDH (U/L)	Stimulated salivary LDH (U/L)
Serum LDH (U/L)	r = 0.337; *P* = 0.001*	r = 0.384; *P* = 0.000*
Unstimulated salivary LDH (U/L)		r = 0.884; *P* = 0.000*

Data were analyzed by the Pearson correlation coefficient

*A significant correlation at *P* < 0.05

### Sensitivity and specificity by the ROC curve

The ROC curves for serum LDH, unstimulated salivary LDH, and stimulated salivary LDH levels were drawn.

In comparison between OLR and control groups, a serum LDH level of 53 U/L had a sensitivity of 77% and a specificity of 84% for detection of OLRs (*P* > 0.05). In comparison between OSCC and control groups, a serum LDH level of 164 U/L had a sensitivity of 88% and a specificity of 100% for detection of OSCC. The area under the curve for serum LDH was 0.962 with a two-tailed *P* < 0.000.

In comparison between OLR and control groups, an unstimulated salivary LDH level of 4 U/L (since this value was < 5 which was the sensitivity limit of this marker, the sample size was doubled so that this value could be measured) had a sensitivity of 94% and a specificity of 67% for detection of OLRs. The area under the curve for unstimulated salivary LDH was 0.854 with a two-tailed *P* < 0.000. In comparison between OSCC and control groups, an unstimulated salivary LDH level of 8 U/L had a sensitivity of 88% and a specificity of 83% for detection of OSCC. The area under the curve for unstimulated salivary LDH was 0.911 with a two-tailed *P* < 0.000.

In comparison between OLR and control groups, a stimulated salivary LDH level of 5.5 U/L had a sensitivity of 96% and a specificity of 75% for detection of OLRs. The area under the curve for stimulated salivary LDH was 0.874 with a two-tailed *P* < 0.000. In comparison between OSCC and control groups, a stimulated salivary LDH level of 8 U/L had a sensitivity of 96% and a specificity of 79% for detection of SCC. The area under the curve for stimulated salivary LDH was 0.952 with a two-tailed *P* < 0.000.

## Discussion

According to the current results, the salivary and serum levels of LDH in OSCC patients were significantly higher than the corresponding values in the OLR, OLP and control groups. Moreover, this study was the first to measure the LDH level in patients with OLRs to assess the risk of malignant transformation of these lesions. The serum level of LDH in the OLR group was significantly higher than that in the OLP and control groups. Also, a significant correlation was noted between the salivary and serum levels of LDH. According to the ROC analysis, LDH could be regarded as an appropriate biomarker for differentiation of OSCC from the control, and OLRs from the control groups.

LDH catalyzes the conversion of pyruvate to lactate and vice versa, as well as the conversion of NADH to NAD+ and vice versa [2]. The LDH level of each tissue may vary depending on its metabolic requirements. The LDH levels may change during the process of growth and development, due to biological changes, and in response to pathological conditions [3]. LDH is released upon the destruction of cell membrane. Thus, measurement of LDH can estimate the rate of cell death, necrosis, and tissue injury in different diseases [33]. Malignant tumor tissue or the contiguous tissue damaged by the tumor often release LDH into the blood stream, which abnormally raises the serum level of LDH [29, 30]. This finding has been confirmed in many cancer types [34–36]. In line with the current findings, several studies have reported increased serum level of LDH in oral cancer [29, 37, 38]. Increased serum level of LDH has also been reported in many premalignant oral lesions. Pereira et al. found increased serum levels of LDH in patients with oral cancer, leukoplakia, and oral submucosal fibrosis (OSMF) by auto-analyzer and spectrometry [39]. Kallali et al. used semi-automatic analyzer and reported significant increase in serum level of LDH in patients with OSMF and oral cancer [2]. Rathora et al. reported a significant increase in serum level of LDH in OSCC, OLP and OSMF groups, compared with the control group and added that assessment of serum level was a less invasive diagnostic method compared with biopsy [29].

The increase in dysplastic changes from a normal tissue to a malignant tissue triggers a shift to anaerobic glycolytic pathway [30]. The LDH activity is mainly due to an increase in mitotic index and further production of lactic acid by tumoral cells due to glycoprotein breakdown in the process of malignant changes. Greater dysplastic or malignant changes would further elevate the level of LDH [40]. Thus, variable levels of LDH in normal, potentially malignant, and malignant groups can be related to different dysplastic and histological grades.

In our study, the serum level of LDH in the OLR group was significantly higher than that in the OLP and control groups. Since this study was not a prospective study, and these lesions have the lowest rate of malignant transformation compared with other oral premalignant lesions, further studies are required to assess the role of LDH in malignant transformation of OLRs. However, histopathological differences between OLP and OLRs, including infiltration of different inflammatory cells, more perivascular inflammation, higher mitotic index and basement membrane cleft in OLRs than OLP [41] and more Civatte bodies due to cell membrane degeneration [12] can justify the higher levels of LDH in OLRs than OLP. According to the literature, the prevalence of malignant transformation is 0.1–5.3% in OLP and 0.5–6.5% in OLRs [20]. According to a new classification by Sarode et al. OLRs are premalignant lesions under group 2b, which is a carcinogenic group due to chronic mucosal inflammation as the result of external stimuli [42]. The process of malignant transformation of OLRs is related to field cancerization. In this process, all related events in such patients expose them to higher risk of primary malignancy [20].

In this study, despite the fact that OLP is a premalignant lesion, the LDH level in this group was not significantly different from that in the healthy control group; whereas, Rai et al. discussed that the level of LDH isoenzymes significantly increased in the OLP group, compared with the control group, which can justify the premalignant nature of OLP [28]. Since different clinical types of OLP have different risks of malignant transformation, difference in distribution of OLP types in the study by Rai et al. can explain the variability in the reported results. The suggested hypotheses for OLP malignancy include increased levels of inflammatory cytokines and growth factors and oxidative destruction of DNA due to chronic inflammation, presence of Candida albicans in OLP, use of immunosuppressive or immunoregulatory agents, and increased lipid peroxidation [20].

Of all body fluids, serum has been the medium of choice for assessment of biomarkers. However, the salivary LDH profile can almost reflect the condition of oral mucosal epithelium (but not salivary glands), which indicates that the main source of salivary LDH is probably the oral mucosal epithelial cell shedding. Thus, assessment of salivary LDH can serve as an efficient tool for evaluation of oral conditions such as oral dysplasia and cancer that compromise the integrity of oral mucosa [42].

Shipter et al. in studies conducted in 2007 and 2009 demonstrated a complete change in the composition of saliva in oral cancer patients. They reported alterations in parameters such as matrix metalloproteinases 2 and 9, IGF-1, and sIgA in the saliva and demonstrated a significant increase in salivary LDH level of oral cancer patients, which was in agreement with the current results [43, 44]. Samlin et al. showed that salivary levels of LDH significantly increased in oral premalignant and malignant lesions [45]. Shetty et al. reported a significant increase in LDH level in males compared with females, and in leukoplakia and oral cancer compared with the control group [22]. Similar results were reported by another study conducted in 2014 on unstimulated saliva samples of leukoplakia and oral cancer patients for estimation of LDH levels using gel electrophoresis [30]. However, in this study, females with OSCC showed higher levels of LDH than males. In 2015, Patel et al. reported a significant increase in LDH level of oral cancer and leukoplakia patients compared with the control group using semi-automatic analyzer [21].

Considering the results of the present study and relatively higher levels of LDH in the saliva of OLR patients compared with controls (non-significant), replacement of serum analysis with saliva analysis requires further investigations. Review of the relevant literature revealed that the correlation of salivary and serum levels of LDH has been rarely studied. Sivaramakrishnan et al. reported increased salivary and serum levels of LDH using a specific kit in OSMF patients but did not find a positive correlation between its salivary and serum levels [46]. Joshi et al. reported a significant increase in salivary and serum levels of LDH in leukoplakia and OSCC patients using a span kit but found no serum-saliva correlation in this respect [3]. Rao et al. used spectrophotometry and showed that the salivary and serum levels of LDH in OSCC patients were significantly higher than the corresponding values in the control group, and the salivary level of LDH was significantly higher than its serum level in both groups. They also found a significant correlation between the salivary and serum levels of LDH in the two groups. Moreover, they showed that the salivary level of LDH was correlated with the frequency and duration of tobacco use in OSCC patients while no such a correlation with serum level of LDH was noted [31]. Despite the fact that the findings of Rao et al. were similar to our results, our study showed higher serum level of LDH than its salivary level in all groups, which is justifiable considering the many sources of serum LDH compared with salivary LDH.

Measuring the total level of LDH and its isoenzymes is important in detection of cancerous and potentially malignant lesions, and can also serve as an important prognostic tool [38]. Some authors, in agreement with the results of the present study, have confirmed that increased serum level of LDH is positively correlated with the histological grade of OSCC [21, 39]. Josh et al. also found a significant correlation between the histological grade of OSCC and salivary levels of LDH isoenzymes [30]. However, this finding was not confirmed by Langvade et al. who measured the tissue level of LDH [47]. Naphade et al. demonstrated that increased density of LDH 2,3,4 was strongly suggestive of higher malignant transformation of oral potentially malignant lesions [38].

In our study, similar to earlier investigations, OSCC patients showed higher serum and salivary levels of LDH. To the best of the authors’ knowledge, this study is the first to show higher level of LDH in patients with OLRs compared with the control and even OLP groups, which can further confirm the highly malignant potential of OLRs. Thus, accurate diagnostic clinical and histological criteria, appropriate treatment planning, and regular follow-ups are strongly recommended for such patients to prevent malignant transformation. Further studies with larger sample size on serum, saliva and tissue are required to measure the exact levels of LDH isoenzymes in patients with different conditions over long-term follow-ups.

## Conclusion

Patients with OSCC and OLRs had higher serum levels of LDH compared with OLP and control groups. We recommend further prospective longitudinal studies especially for assessment of the tissue levels of lactate dehydrogenase to assess the transformation of OLRs because their malignant transformation is low compared with other oral premalignant lesions.

## Data Availability

The dataset used and/or analyzed during the current study are available from the corresponding author on reasonable request.
